# The Evolution of the Role of Imaging in the Diagnosis of Craniosynostosis: A Narrative Review

**DOI:** 10.3390/children8090727

**Published:** 2021-08-25

**Authors:** Giovanni Cacciaguerra, Monica Palermo, Lidia Marino, Filippo Andrea Salvatore Rapisarda, Piero Pavone, Raffaele Falsaperla, Martino Ruggieri, Silvia Marino

**Affiliations:** 1 Department of Clinical and Experimental Medicine, Section of Pediatrics and Child Neuropsychiatry, Unit of Rare Diseases of the Nervous System in Childhood, University of Catania, 95125 Catania, Italy; gio.cacciaguerra@gmail.com (G.C.); ppavone@unict.it (P.P.); m.ruggieri@unict.it (M.R.); 2 Radiology Unit 1, Department of Medical Surgical Sciences and Advanced Technologies “GF Ingrassia”, University of Catania, 95125 Catania, Italy; monica.palermo91@gmail.com; 3 Neonatal Intensive Care Unit, AOU “Policlinico”, PO “San Marco”, University of Catania, 95121 Catania, Italy; lidia.m@hotmail.it (L.M.); raffaelefalsaperla@gmail.com (R.F.); 4 Department of Obstetrics and Gynecology, ARNAS Garibaldi Nesima, 95122 Catania, Italy; filipporapisarda83@gmail.com; 5 Unit of Pediatrics and Pediatric Emergency, AOU “Policlinico”, PO “San Marco”, University of Catania, 95121 Catania, Italy

**Keywords:** craniosynostosis, sutures, imaging, children, neurodisability, plagiocephaly

## Abstract

Craniosynostosis, the premature closure of cranial sutures, is one of the principal causes of pediatric skull deformities. It can cause aesthetic, neurological, acoustic, ophthalmological complications up to real emergencies. Craniosynostosis are primarily diagnosed with accurate physical examination, skull measurement and observation of the deformity, but the radiological support currently plays an increasingly important role in confirming a more precise diagnosis and better planning for therapeutic interventions. The clinician must know how to diagnose in the earliest and least invasive way for the child. In the past, technological limitations reduced the choices; today, however, there are plenty of choices and it is necessary to use the various types of available imaging correctly. In the future, imaging techniques will probably rewrite the common classifications we use today. We provide an updated review of the role of imaging in this condition, through the ages, to outline the correct choice for the clinician for an early and non-invasive diagnosis.

## 1. Introduction

Craniosynostosis, also known as cranial synostosis, craniostenosis, or, simply, synostosis, refers to a congenital condition characterized by the premature closure of one or more cranial sutures. This process leads to characteristic skull deformities, facial asymmetry, and impairment of brain development [1]. “Single” synostosis, when premature closure affects one cranial suture, or “compound” synostosis, affecting more than one sutures [2,3], may occur as a primary condition (isolated or in a syndromic form), or secondary to various underlying causes, such as metabolic, intracranial, teratogenic or hematological conditions [4,5].

Craniosynostosis affects male children more frequently than females (4:1), and it often occurs already at birth, but it becomes increasingly noticeable during the first months of life as a cranial deformity [4,5].

Plagiocephaly represents the first cause of pediatric head deformities, but it is rarely caused by a premature closing of lambdoid suture, being more often caused by a non-siynostotic cause—known as deformational plagiocephaly. The most commonly affected site of craniosynostosis is sagittal suture (40–60% of cases) [3], followed by coronal synostosis.

The first diagnosis of typical cases of craniosynostosis is usually clinical, and the inspection should determine whether a synostosis is present, which type, whether there are any features suggesting a syndromic form and whether and which management is required [1]. Even though diagnosis can be made after a clinical evaluation, it may be challenging to establish the extent of cranial involvement by clinical examination alone, and the diagnosis is usually confirmed by radiological examinations, especially in compound synostosis and when a surgical treatment is planned [1,6]. Imaging is indeed essential for an accurate diagnosis, operative planning, post-surgical evaluation and identification of coexisting anomalies and complications [7]. Radiology initially provided support in the diagnosis with plain radiographies: in 1948, D. Fairmand and G. Horrax in their paper *Classification of Craniostenosis* stated that “it is indispensable to have x-ray evidence of the premature closure of the sutures” [8]. Plain films can certainly show signs of synostosis and provide useful information, but computed tomography (CT) with three-dimensional (3D) reconstructions, which is considered the most complete and accurate imaging to diagnose craniosynostosis [9] have overtaken them. Clearly, the use of ionizing radiations and the risk of late radiation effects are of major concern and lead to look for diagnostic alternatives [10], but CT scans may be unavoidable in complex, syndromic or complicated types of craniosynostosis for the best treatment planning [7]. The advent of MRI opened up new possibilities for studying brain anomalies associated with craniosynostoses and, thanks to technological progress, for recognizing and distinguishing cranial bones from cranial sutures. Ultrasound (US) examination is a fast, low-cost, radiation-free method, and it can be considered as a continuation of the clinical examination when performed bedside. US can be also applied for prenatal diagnosis and as a follow-up tool.

### Classification

Except for Hippocrates’ first description in 100 B.C., craniosynostosis were firstly described in the early 19th century by Sömmerring and Otto, and later defined as we know them today by Virchow in 1851. There are different types of craniosynostosis, depending on which suture(s) is involved. The first classification system was published by Virchow in 1851, based on four major types of head shape: macrocephaly (large head), microcephaly (small head), dolichocephaly (long head) and brachycephaly (short head). Virchow correlated head shape with the fusion of specific sutures and introduced some definitions that are still in general usage such as dolichocephaly for sagittal synostosis, trigonocephaly for metopic synostosis, and plagiocephaly for unilateral coronal synostosis. During the first years of the 20th century, familial and hereditary craniofacial dysmorphisms were described, firstly by Apert in 1907 and Crouzon in 1912, who coined different terms and introduced an eponymic classification system; this system remains nowadays in large clinical usage, as it is efficient in describing clinical phenotypes. Many other classifications have been proposed during recent years, especially focusing on molecular pathogenesis, also thanks to the Human Genome Project, which increased knowledge on craniosynostosis’ genetic etiologies [11,12].

## 2. Research Strategy

Through main electronic medical database search (Pubmed, Embase, Scopus, Cochraine Library and Web of Science), we performed a thorough analysis among all the papers about craniosynostosis, with a particular focus on their publication date. We then dated back to the first time this topic was covered, and we focused our attention on the diagnostic tools every article mentioned. After this meticulous investigation, we suggested three different “eras” of imaging in craniosynostosis, each one characterized by a predominating diagnostic tool or by the introduction of a new technology that drastically changed the diagnostic status quo.

## 3. Past (1950–1980): Not Only the Physical Exam

In 1933, Harry Greene, in his paper “Oxycephaly in man and rabbit” [13], describes and compares cranial deformities in rabbit and man, stating that, in rabbits, “casual observation would not distinguish affected animals from normal members and although close examination may separate a shortened or asymmetrical head, correct differentiation can only be made by careful palpation” [13]. There are not any references to imaging techniques within the text. The first radiographic studies were encountered in the work of A.L. Peter, Maj [14] in 1946, presenting four cases of mild oxycephaly in soldiers. X-rays are described as diagnostic for revealing skull anomalies and associated deformities in the spine (such as the absence of cervical vertebrae in Klippel–Feil syndrome) or in the hands (such as deviation or arachnodactyly) [14]. In 1945, a “roentgenographic examination” of a child with acrocephalosyndactyly (Apert syndrome) was performed [15], demonstrating synostosis of the coronal suture, lacunas on the vault, dimensional anomalies of anterior, middle, and posterior fossa, and the presence of syndactyly. Over the years, x-ray examination became increasingly more common and more important in the diagnosis and classification of craniostenosis, as stated by Fairman in 1948 [14]: “The x-ray examination enables us to determine with accuracy the variety of craniostenosis and the degree of contraction of the skull”; moreover, they were used in order to perform diagnostic tests, such as ventriculography, and post-surgical studies [10]. Many papers, describing cases of different types of craniosynostosis, classifications and treatment, showed the application of radiological imaging in diagnostic management [16,17,18,19,20]. Radiological examinations were used also in case of secondary craniosynostosis, for example in a case report about a case of vitamin D-resistant rickets needing a decompressive craniotomy [19]. X-rays showed classical signs of rickets in wrists, limbs and thorax, and the skull presented with increased impressions and a prominence in the sagittal plane, the coronary suture was hardly visible, and the other sutures were small. Preoperative assessment with x-rays became ever more used: in 1965, Anderson et al. published a survey of 204 cases, highlighting the “importance of precise clinical and radiological diagnosis and proper surgical management” [20]. With the advancement of technologies, new ideas have been proposed to be applied in this field: in 1975, Gates and Dore presented the results of their studies using bone scanning with 18G or 99mTc-poliphosphate to ameliorate clinical and radiographic detection of suspected craniosynostosis [21]. Scans disclosed abnormal and nonuniform radionuclide distribution along a prematurely closing suture, with focal osteoblastic hyperactivity; accumulation diminished when suture fusion was complete. Some years later, Tait et al. demonstrated patterns of sutural activity in their interesting correlation of bone scans, radiographs and surgical findings [22]. Their results revealed that scans can be of great assistance in case of abnormal or equivocal x-rays, but when the radiography was normal, the scans have a little contribution. Therefore, the radiograph remains the first investigation in a child with suspected craniosynostosis, and if it is normal, the scan is probably unnecessary. By the end of the 1970s, Rasheed U. Adam [23] had found some criticism on x-rays and bone scans: skull radiographs can show falsely patent suture and radionuclide bone scan can show paradoxical uptake and has low reliability after surgery. In this context, CT scans were proposed, by far, as the single most important study “because it not only affords superior visualization of the status of sutures and head shape but also clearly demonstrates any associated intracranial abnormalities”. The CT study of the parenchyma changed the obsolete concept that single craniosynostosis was only a “cosmetic problem” that had no affection on brain [24]. Since then, CT has taken over plain radiographies, obtaining great positive responses thanks to its diagnostic accuracy on bones and parenchyma.

## 4. Recent Times (1980–2010): Cranial Suture Ultrasound Age

**Figure 1:**
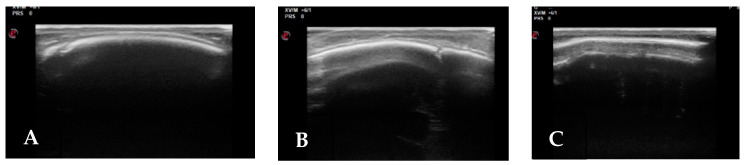
Suture US: (**A**) Patent right coronal; (**B**) Patent right lambdoid; (**C**) Synostotic sagital.

**Figure 2:**
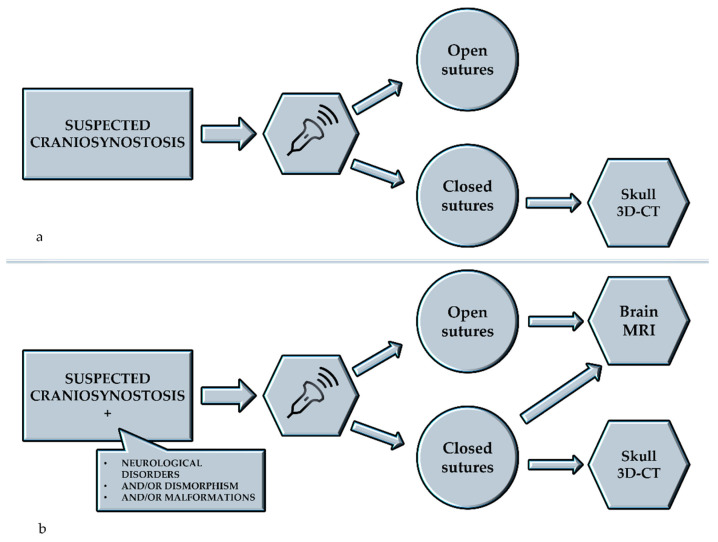
Management of cases of suspected craniosynostosis starting with ultrasonographic examination: (**a**) suspected craniosynostosis alone; (**b**) suspected craniosynostosis associated with other conditions. 3D-CT: three-dimensional computed tomography, MRI: Magnetic Resonance Imaging.

Sobolesky et al. proposed the ultrasound (US) examination for the study of cranial sutures for the first time in 1997 and 1998 [25,26]. About 10 years later, two papers published by a neurosurgical team from Hamburg described the use of US in the diagnosis of craniosynostosis and positional plagiocephaly [12,27]. In the first study [12], the authors compared the imaging of the synostotic sutures of 26 infants (2–7 months) obtained with US and CT. They described how all 26 patients with partial (*n* = 21) or total (*n* = 5) fusion of one or more sutures could be reliably diagnosed by US. In the same year, they studied 100 infants (2–13 months) with positional plagiocephaly. Patency of lambdoid sutures was confirmed by ultrasound in 99 cases, in which clinical results suggested positional palagiocephaly. Other authors have described good rates of sensitivity and specificity of US in the diagnosis of craniostenosis [28,29]. This technique (Figure 1) is simple and non-invasive, it does not require preparation or sedation; it appears to be a useful radiation-free screening tool to obtain an early diagnosis, to make differential diagnosis with microcephaly and it is also well tolerated by patients and parents. However, this method comes into difficulty in routine clinical practice, for several reasons. As is well-known in the scientific community, US is the most operator-dependent imaging technique. The physician who performs the US needs to be very experienced in the field, in order to perform a technically perfect examination, ensuring that the entire length of the suture is visualized, and to correctly interpret the imaging. Moreover, especially in cases of suspected craniostenosis associated with other conditions, US is usually followed by other studies such as brain MRI (in case of opened sutures, to study the brain) and skull 3D-CT, to confirm the US diagnosis (Figure 2).

Furthermore, US can be used as a prenatal diagnostic technique: severe cranial malformations can be recognized during the third trimester of pregnancy.

The prenatal US diagnosis of craniosynostosis may also be hard for experienced sonographers, and it is usually performed when there is a reasonable suspicion such as abnormal cephalic index, cranial shape or fetal face or when a genetic syndrome is suspected [23]. Prenatal US represents a safe examination to suggest the diagnosis of craniosinostosis; it will be usually subsequently confirmed by MRI, which can accurately detect brain abnormalities, which are an important predictive factor.

## 5. The Computed Tomography

**Figure 3:**
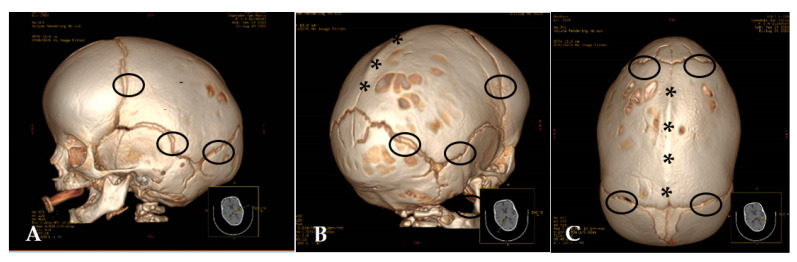
(**A**–**C**) CT 3D-Reconstructions (**A**) Scaphocephaly with patent sutures (ring); (**B**,**C**) Patent sutures (ring) and synostotic sagital suture (asterisks).

In these decades, the role of CT in the diagnosis of craniosynostosis grew and became increasingly fundamental; in fact, countless scientific publications affirm that the diagnosis of craniosynostosis relies on physical examination and radiographic studies, including plain radiography and computed tomography (CT) [3]. In 1981, Carmel P.W. et al. [24] stated that plain roentgenography has been the traditional method for evaluating the deformities of craniosynostosis, but that the advent of CT “has permitted simple and easy evaluation of both the base changes and the vault deformities”. Initially, only axial CT sections were available, and they could be deficient for some kinds of synostosis, such as the sagittal kind. [24]. Later, more modern scanners could enable to perform reconstructions and reformatted planar views, to choose the better plan to demonstrate the anomaly. Most cranial sutures are best-assessed using 3D reconstructions, as these images provide information that cannot be appreciated on axial 2D CT or plain films, obtaining a diagnostic accuracy approaching 90–100%. Fist works about 3D reconstruction from CT scans date back to the early 1980s, and its first application on craniofacial field was presented by J.L. Marsh et al. in 1983 [30], soon followed by many other physicians [31,32,33,34]. CT scan with 3D-reconstruction provides, at the same time, information regarding structural brain, craniofacial, and skull base anomalies [34]. The development of multi-detector row CT (MDCT) allows a substantial reduction in examination time for standard protocols, coverage of extended anatomic volumes, substantially increased longitudinal resolution employing reduced section width, and high-resolution volumetric data to be obtained [35] (Figure 3).

In these decades, the use of CT, although widely accepted, is also criticized for radiation doses and for its lifetime risk of fatal cancer. For this reason, radiological techniques that do not emit radiations are, also nowadays, deepened and have a relevant place in scientific discussion.

## 6. Future (2010–2021): New Applications and Possibilities

Future prospects are based on a multidisciplinary approach: radiologists, pediatricians, geneticists have to cooperate, and technological development refines, every day, what we know and redefines what we can do. Wider and more powerful informatic systems allow medicine to open new horizons.

The diagnostic role of ultrasound study is strengthened: for N. Kajdic et al. [1], standard ultrasonography of the calvarial sutures, in the absence of other craniofacial malformations, may be a feasible method for diagnosing simple, non-syndromic craniosynostosis in utero. An important advantage of ultrasonography is the possibility to visualize and follow-up the cranial sutures on every examination, to obtain the best scan for measuring the skull and one or more prematurely fused sutures. In experienced hands, with this issue remaining as the major limit of its application, US may be as reliable as CT. The diagnosis is possible after the first trimester [1]. A new method for improved prenatal detection of craniosynostosis is the incorporation of 3D sonography, proposed by Ginath et al. [36]. They compared the detection of sutures in anatomically normal fetuses between 15 and 16 weeks of gestation using transvaginal 2D versus 3D imaging. Although there was no significant difference in the number of patients in which all sutures could be identified, identification of the sagittal suture was enhanced in the 3D group compared with the 2D group [36].

In 2020, Brah et al. [37], during a literature review, sharing the same concepts of Ginath et al., stated that both 3D ultrasonography and MRI have been identified as useful modalities to aid detection in high-risk individuals. Prenatal detection can allow families to prepare and practitioners to provide appropriate clinical treatment [37].

The superior imaging quality of CT scans on cortical bones and the possibility of creating 3D reconstructions were one of the major limitations of wider applicability of MRI, but advancements in technologies are working hard to propose MRI as a no-radiation alternative in this field. New protocols, such as black-bone imaging, can provide high-quality 3D imaging of bones and sutures similar to those of CT, while giving, in the same session, details about structural anomalies and prognostic information [38,39]. Eley et al. first reported the black-bone imaging technique in literature in 2012 [40]; thanks to short TE/TR and low flip angle, it makes it possible to minimize soft-tissue contrast to enhance the bone-soft tissue interface. Patent sutures have a different signal intensity than prematurely fused ones [41]. Moreover, it is possible to provide 3D-reconstructed images and, also, to create 3D models using a 3D printing [39,41].

New further development in functional imaging could provide useful information about the underlying microstructural changes in these patients, studying neural plasticity, compensatory development, behavioral, attentional, and emotional sequelae and differences in network connections and neurological changes before and after treatment [42,43].

## 7. Conclusions

It is common knowledge that diagnosis is often a network of ideas and findings, and that the physician’s critical thinking is increasingly guided by an approach that must be as unaggressive as possible. It is certainly very stimulating to see how the evolution of technology can always provide new food for thought. Since the beginning of medical thought, the eye has been the clinical tool par excellence; through the years, technologies have changed our way of seeing and of interpreting. Of course, technological advancement cannot be the only reason to change and evolve diagnostic choices: a close multidisciplinary collaboration is always fundamental in the management of complex conditions, such as the examined one. Neurodisabilities are conditions of increasing interest for different categories of physicians and specialists, and the paediatrician has the important role of coordinating the various medical figures and offering the best options to the patient and his family. Thus, the diagnostic investigation in craniosynostosis has been in continuous evolution. It is truly exciting to experience a period of medical era so inspirational and full of opportunities.
